# Circulating Monocyte Chemoattractant Protein-1 (MCP-1) in Patients with Primary Biliary Cholangitis

**DOI:** 10.3390/ijms25021333

**Published:** 2024-01-22

**Authors:** Alicja Bauer, Tomasz Rawa

**Affiliations:** 1 Department of Biochemistry and Molecular Biology, Centre of Postgraduate Medical Education, Marymoncka 99/103, 00-022 Warsaw, Poland; 2 Department of Gastroenterology, Hepatology and Clinical Oncology, Centre of Postgraduate Medical Education, Roentgena 5, 02-781 Warsaw, Poland; tomasz.rawa@nio.gov.pl

**Keywords:** primary biliary cholangitis, MCP-1, liver fibrosis, autoantibodies

## Abstract

Primary biliary cholangitis (PBC) is a chronic autoimmune liver disease that leads to the destruction of the intrahepatic bile ducts. While the inflammatory process can be mediated by monocyte chemotactic protein-1 (MCP-1), the importance of circulating MCP-1 as a biomarker is unclear. Our aim was to assess the diagnostic significance of the serum concentrations of MCP-1 in PBC patients. We compared circulating MCP-1 with biochemical, immunological and histological parameters. Serum samples were collected from 120 PBC patients, 60 pathologic controls and 30 healthy donors. MCP-1 levels were determined by using commercial enzyme-linked immunosorbent assay (ELISA). Elevated serum MCP-1 levels were detected in 66% of PBC patients with a specificity of 97%. Significantly higher levels of MCP-1 protein were found in the sera of patients with PBC than in the group of healthy individuals—410.2 pg/mL vs. 176.0 pg/mL, *p* < 0.01). Patients with higher concentrations of alkaline phosphatase also had higher levels of MCP-1 (r = 0.4, *p* < 0.01). In accordance with Ludwig’s classification, a positive correlation of serum MCP-1 concentration with the degree of fibrosis was observed, OR = 6.1, *p* = 0.0003. We compared the MCP-1 with procollagen type III, hyaluronic acid (HA), FIB-4 index, APRI and collagen type IV when predicting the advance of liver fibrosis. Circulating MCP-1 is better correlated with liver fibrosis and is also associated with the occurrence of specific antimitochondrial autoantibodies and specific anti-nuclear autoantibodies—anti-gp210. MPC-1 can be considered to be a tool for diagnosing the degree of fibrosis in PBC, and combinations of MCP-1 and other specific biomarkers could support the diagnosis of PBC.

## 1. Introduction

Primary biliary cholangitis (PBC) is an autoimmune, cholestatic liver disease that is associated with the progressive destruction of the bile ducts, which leads to fibrosis, and ultimately to cirrhosis of the liver [1,2,3]. The diagnosis of PBC is based on clinical, biochemical, and radiological markers of intrahepatic cholestasis [4,5,6]. More than 90% of patients have circulating antimitochondrial autoantibodies (AMAs) [7,8], and about 30% to 50% of patients with PBC also have antinuclear antibodies, including anti-gp210, anti-p62 and anti-sp100. These anti-nuclear autoantibodies are associated with severe disease and poor outcomes [1,2,9,10,11,12,13,14,15]. The inflammatory and destructive process leads to advanced fibrosis, cirrhosis, and liver failure. Liver inflammation can be regulated by matrix metalloproteinases [16,17] and different chemokines [18]. Monocyte chemoattractant protein-1 (MCP-1) is one of the most convincing profibrogenic chemokines in the development of liver fibrosis [19], which belongs to the C subfamily of chemokines, is characterized by monocyte chemotactic activity [20] and has also been recognized as a significant chemotactic mediator of monocytes/macrophages. Recent studies have also shown that MCP-1 can chemoattract T lymphocytes and endothelial cells [21,22], and has a significant function in the pathogenesis of various diseases [23,24,25,26,27,28,29]—the role of MCP-1 has been investigated in the pathogenesis of interstitial lung disease, breast, prostate cancer, and cytomegalovirus encephalitis [24,30]. Several studies have also previously described its profibrotic role in autoimmune diseases, such as rheumatoid arthritis and systemic sclerosis [31,32,33,34]. It has been observed that its profibrogenic and inflammatory responses played a fundamental role in the development of some hepatic disorders, such as alcoholic liver disease and hepatic fibrosis [19,35,36,37]. Some studies have confirmed higher circulating MCP-1 in patients with nonalcoholic steatohepatitis (NASH) or non-alcoholic fatty liver disease (NAFLD), when compared to healthy controls [36,38]. MCP-1 is also involved in inflammation and fibrosis response to the development of pulmonary fibrosis [29]. As MCP-1 is related to different pathological conditions, various reports also suggest that MCP-1 can be a tool to evaluate the intensity of inflammation in several diseases [20,21]. Circulating MCP-1 comes from a damaged liver and may be linked to the severity of liver disorder [39].

The aim of our study was to evaluate the concentration of MCP-1 in the blood serum of patients with PBC, and compare them with biochemical, immunological, and histological parameters, in order to determine its prevalence and clinical significance in PBC.

## 2. Results

### 2.1. Clinical, Histological and Laboratory Features of PBC Patients

We summarized the clinical, histological, and laboratory data of PBC patients and healthy adult blood donors in Table 1.

In our PBC group, 116 out of 120 patients were females. The mean age at PBC diagnosis was 51 years. The total bilirubin concentration was elevated in over 55% of the tested patients (normal value < 1.2 mg/dL). We found increased activity of AP and γ-GT in over 70% of the tested samples, and the activity of AST and ALT was also higher in over 60% of them. The normal value for AST was <40 U/L; ALT < 40 U/L; AP < 115 U/mg/dL; γ-GT < 50 U/L; albumin 3.5–5.5 g/dL, γ-globulin < 3 g/dL. AMA M2, specific autoantibodies for PBC, was detected in 87% of patients’ sera and specific anti-nuclear antibodies—anti-gp210 autoantibodies were positive in 39% of patients. All of our patients had a liver biopsy. We found small histopathological changes (grade I or II, according to Ludwig’s classification) in 75 cases (63%), and advanced fibrosis (grade III or IV, according to Ludwig’s classification) was detected in 40 cases (33%).

### 2.2. Occurrence and Diagnostic Value of MCP-1

In the tested PBC patients, an elevated concentration of MCP-1 was observed in 79 out of all 120 samples (66%). In the healthy control group, an abnormally elevated level of MCP-1 was found in only one subject out of 30 (3%). We also observed a significant difference between the mean concentration of MCP-1 in the group of PBC patients and the healthy control 410.2 ± 318.0 pg/mL vs. 176 ± 35.5 pg/mL, *p* < 0.0001.

There were only four men in our group—the level of MCP-1 in the serum of men was higher than in the group of women 710.0 ± 350.5 vs. 400.1 ± 314.9, *p* = 0.056, but was not statistically significant.

**Figure 1:**
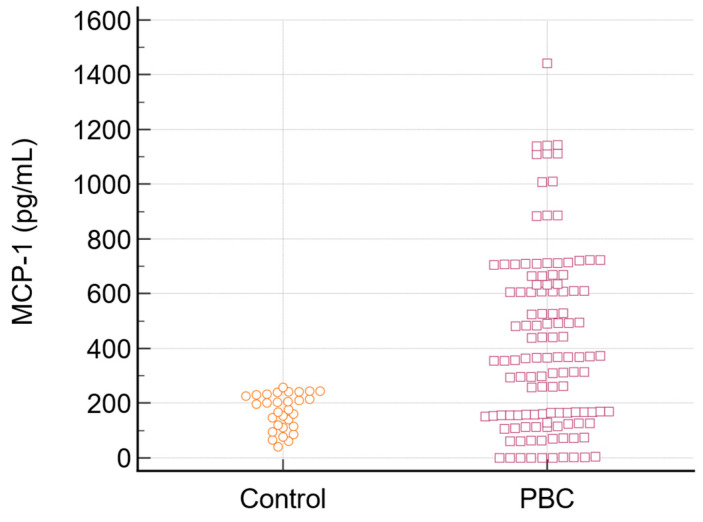
The distribution of MCP-1 in each of the tested groups.

The distribution of MCP-1 in PBC patients and the healthy control group is shown in Figure 1.

**Figure 2:**
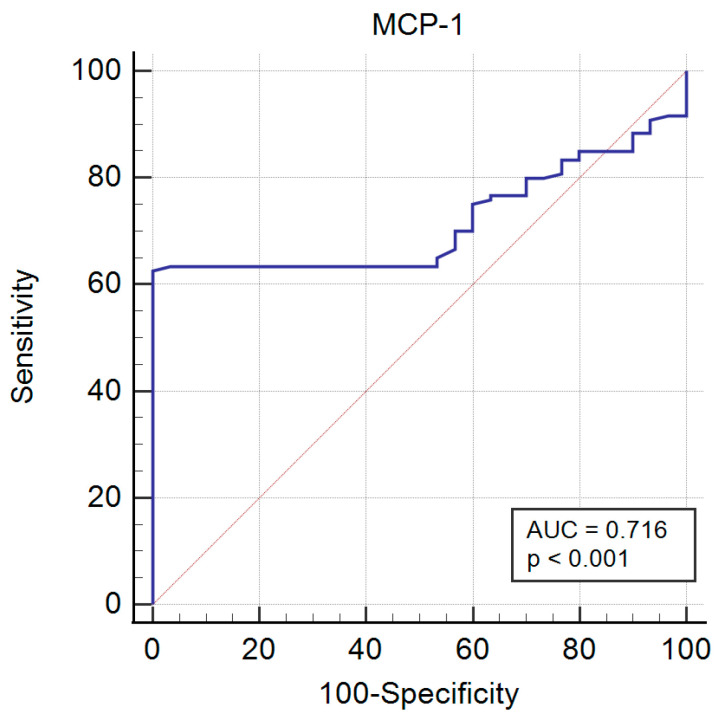
The receiver operating characteristic curve (ROC) analysis for the serological detection of MCP-1 in PBC patients.

The receiver operating characteristic curve analysis for the serological detection of MCP-1 in PBC patients is presented in Figure 2.

AUC = 0.72 for PBC is considered acceptable, and the specificity of serum MCP-1 for PBC was 97%. The positive and negative likelihood ratios were 19.8 and 0.4, respectively, and the calculated positive and negative predictive values were 98.8% and 41.4% respectively.

We detected MCP-1 serum concentration in two other types of liver disease, namely NAFLD and AIH. In the group of NAFLD patients, elevated serum MCP-1 levels were found in 78% (31/40) of cases, and a mean value of 189.0 ± 114.70 pg/mL was recorded, with a significant statistical difference compared to the group of PBC patients, at *p* < 0.001. In the group of patients with AIH, an increased level of MCP-1 in serum was found in 35% of cases (7/20), and the mean value was 273.7 ± 110.3 pg/mL, which was significantly lower than in the group of patients with PBC, but was not significantly statistically different, at *p* = 0.06.

### 2.3. Biochemical and Histological Features of PBC Patients According to the Level of MCP-1

**Figure 3:**
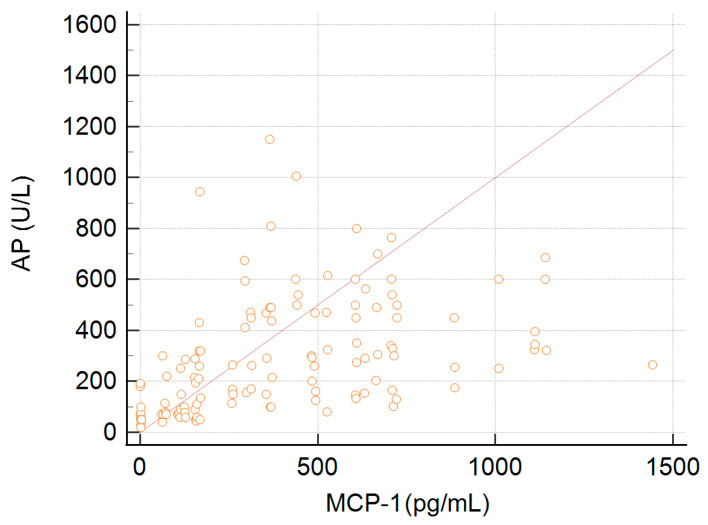
Serum MCP-1 concentration and level of alkaline phosphatase (AP).

We observed an association between the serum concentration of MCP-1 and alkaline phosphatase levels—R = 0.4, *p* < 0.0001 (Figure 3).

The mean concentration of MCP-1 detected in sera of patients with elevated levels of AP was 502.0 ± 302.9 pg/mL, and in patient sera with a normal alkaline phosphatase level, the MCP-1 concentration remained at 126.4 ± 157 pg/mL, *p* < 0.001.

**Figure 4:**
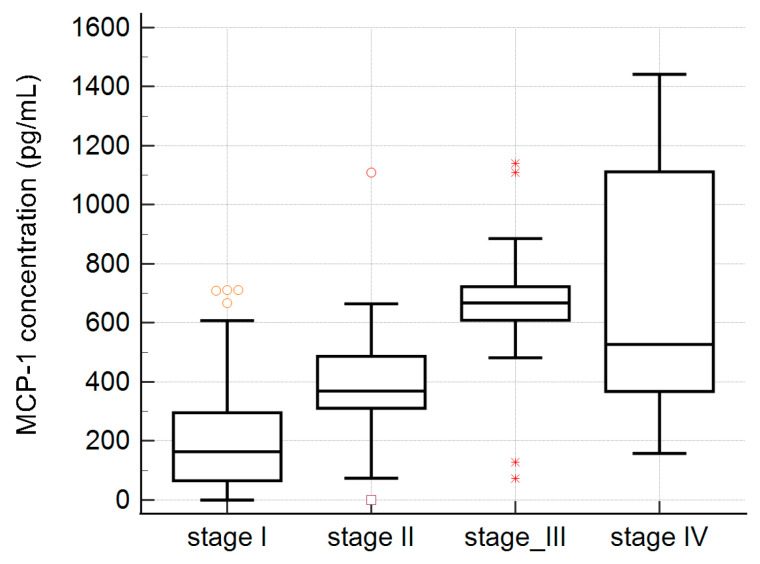
MCP-1 concentration in the sera of PBC patients and the stage of fibrosis (according to Ludwig’s classification).

We compared the concentrations of MCP-1 in the sera of patients with mild changes in liver tissue (stage I or II), and the sera of patients with advanced fibrosis (stage III or IV, according to the Ludwig classification). Mean serum MCP-1 levels in patients with grade I or II fibrosis were 302.8 ±184.2 pg/mL, and mean serum MCP-1 levels in patients with grade III or IV fibrosis were 723 ± 318.0 pg/mL. It was noticed that the serum MCP-1 level demonstrated a statistically significant increase as fibrosis severity increased in PBC patients (*p* < 0.0001). The evaluated OR (95% CI) for the histological score was 6.1 (2.2–17.4), *p* = 0.0006. Among 75 PBC patients with early histological stages (I/II) of the disease, 40 (53%) presented higher levels of MCP-1, in contrast in the group of patients with advanced histological stages (III/IV), of whom 35 out of 40 (88%) tested positive for MCP-1, *p* = 0.0002. In accordance with Ludwig’s classification, we presented MCP-1 concentrations in the sera of PBC patients in four different stages of fibrosis (see Figure 4).

**Figure 5:**
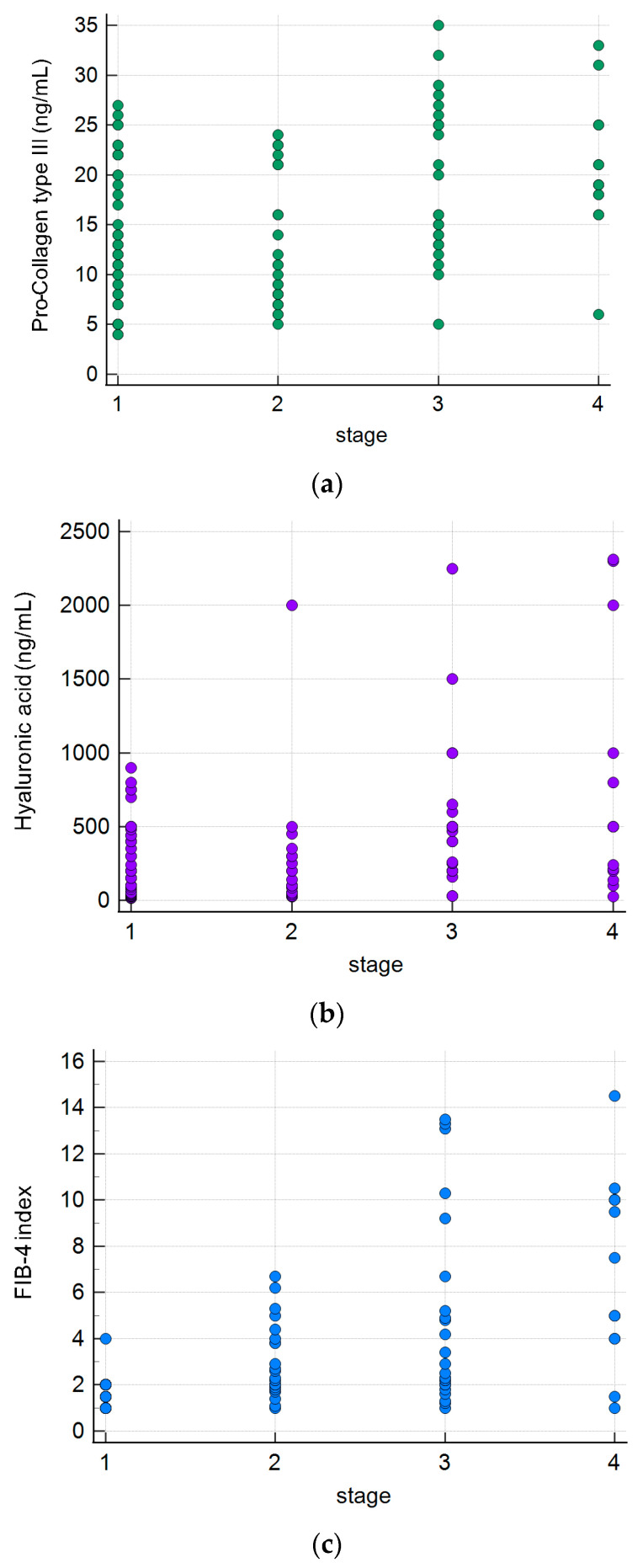

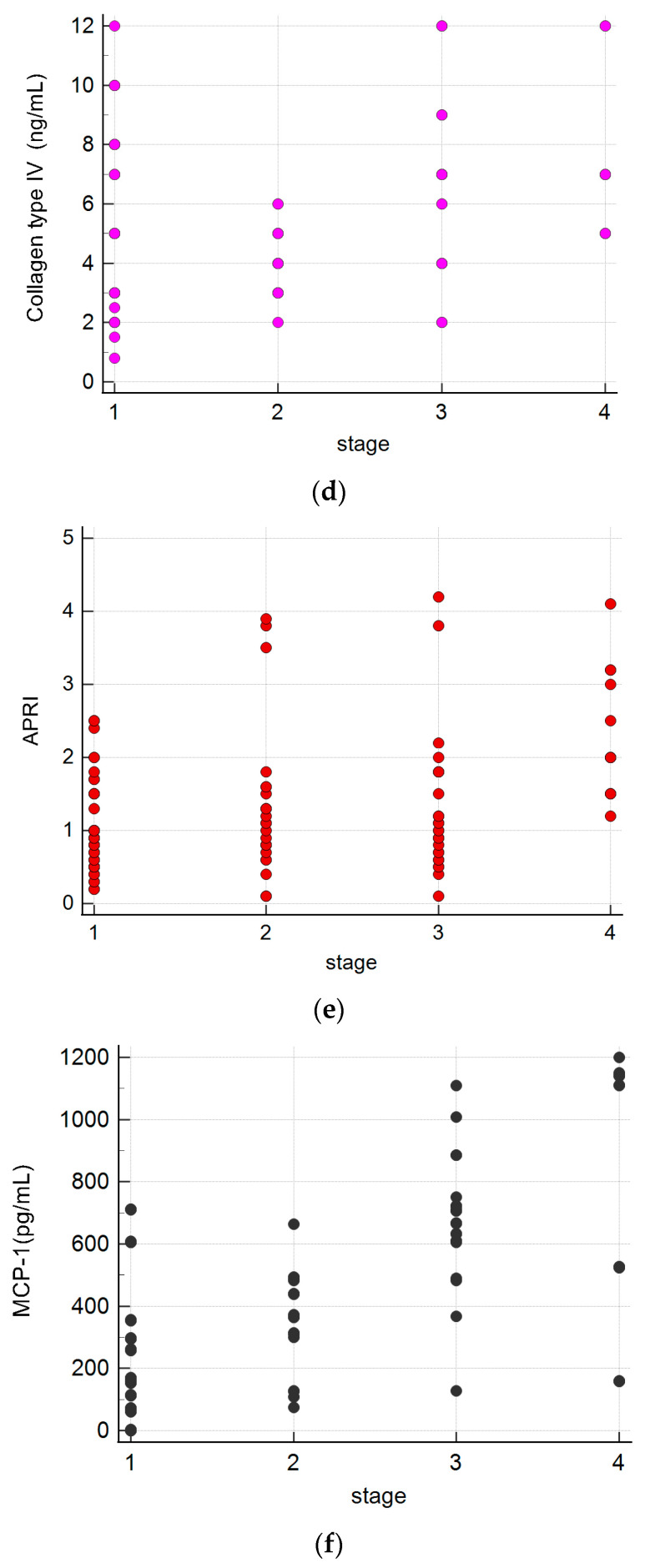
Five liver fibrosis biomarkers, MCP-1 and stage of fibrosis (according to Ludwig’s classification) in patients with primary biliary cholangitis. (**a**) Pro-Collagen type III, (**b**) Hyaluronic acid—HA, (**c**) FIB-4 index, (**d**) Collagen type IV, (**e**) APRI, (**f**) MCP-1.

We compared the five liver fibrosis indices (procollagen type III, hyaluronic acid (HA), FIB-4 index, APRI and collagen type IV) in terms of their ability to predict the advance of liver fibrosis. Mean values and the standard deviation of the procollagen type III, hyaluronic acid (HA), FIB-4 index, APRI and collagen type IV were 15.24 ± 7.52, 366.72 ± 470.08, 3.31 ± 3.14, 1.27 ± 0.89, 5.20 ± 2.71, respectively. We related these indicators to the chemokine MCP-1 in the evaluation severity of liver fibrosis in patients with PBC. Spearman’s rank correlation coefficients between the Ludwig’s stage and procollagen type III level, HA level, FIB-4 index, APRI, collagen type IV and MCP-1 were R = 0.36, *p* < 0.001; R = 0.32, *p* < 0.001; R = 0.59, *p* < 0.001; R = 0.25, *p* = 0.001; R = 0.37, *p* < 0.001 and R = 0.68, *p* < 0.001, respectively, as shown in Figure 5a–f. Circulating MCP-1 showed a better correlation when compared to these methods.

**Figure 6:**
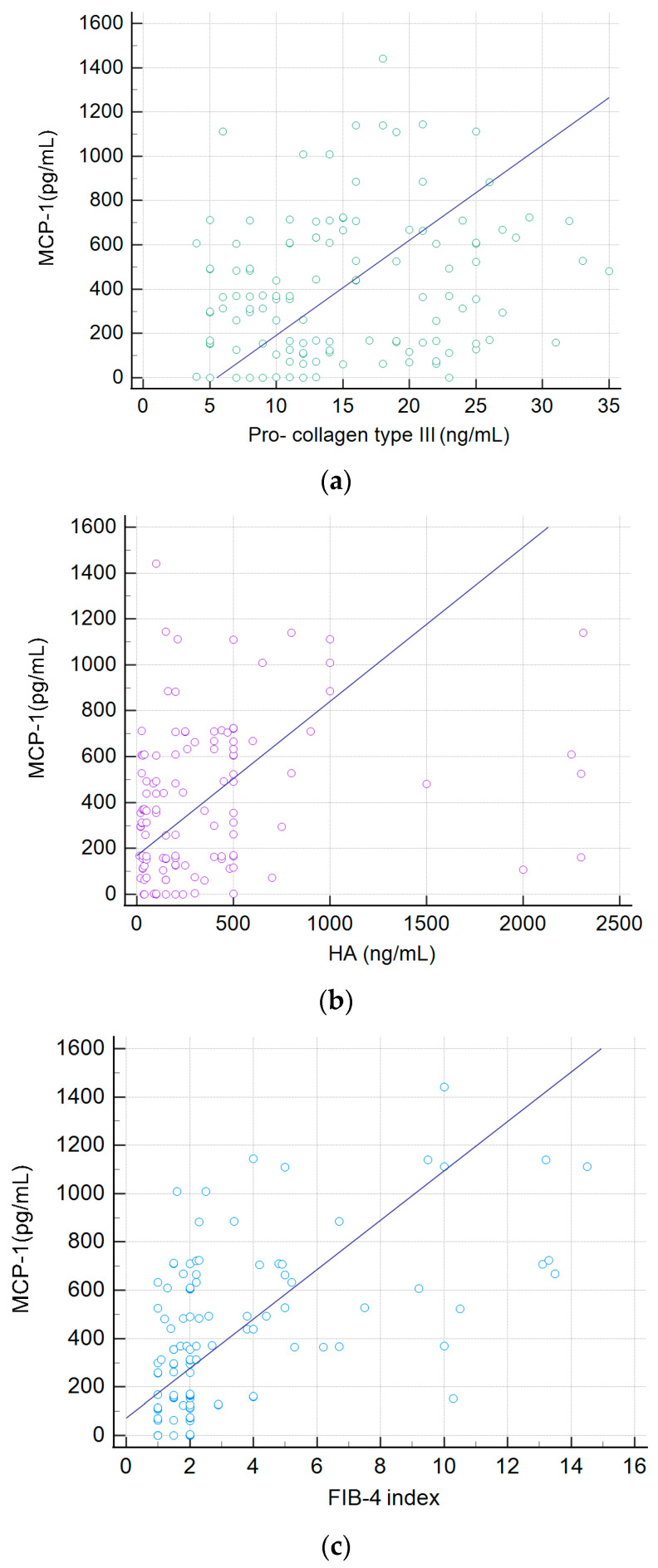

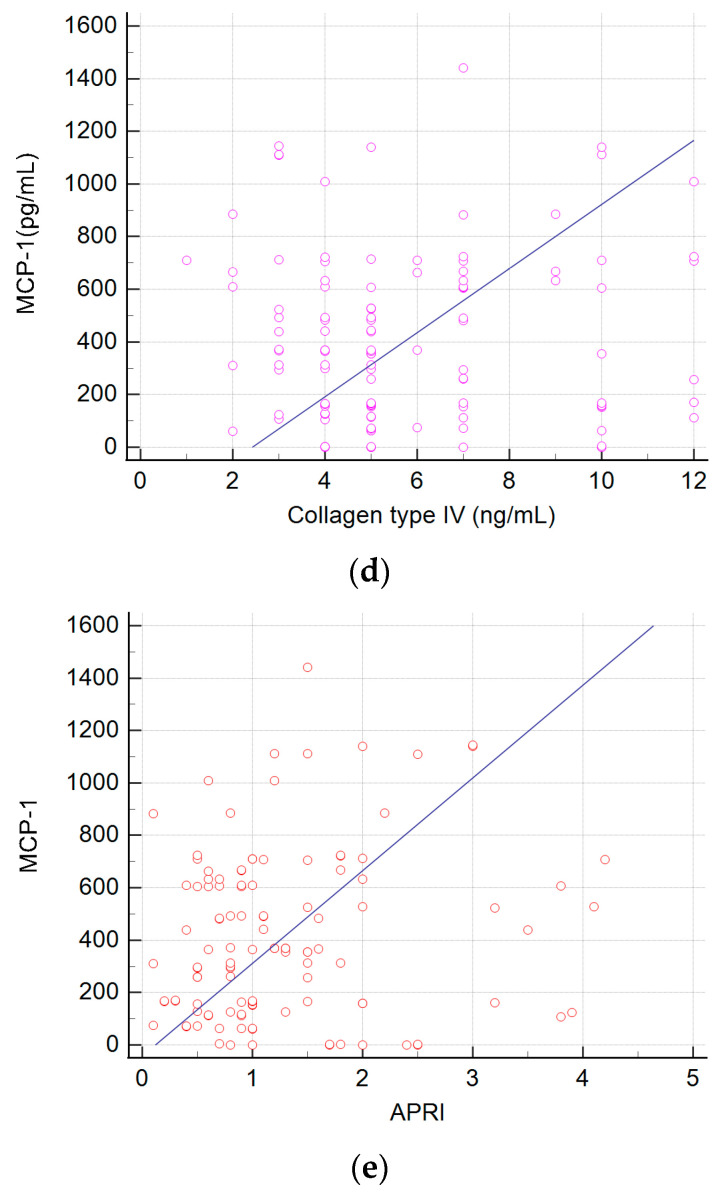
Correlation between serum MCP-1 concentration and level of (**a**) Pro-Collagen type III, (**b**) Hyaluronic acid—HA, (**c**) FIB-4 index, (**d**) Collagen type IV, (**e**) APRI in patients with primary biliary cholangitis.

We evaluated the correlation between the serum concentration of MCP-1 and these fibrosis markers. The results are presented in Figure 6a–e.

We obtained the best correlation for the FIB-4 index, R = 0.51; moderate correlation for HA, R = 0.31, *p* = 0.001; very weak for APRI, R = 0.13 and for Pro-Collagen type III, R = 0.20, *p* = 0.028; and the negative for collagen type IV, R= −0.01.

For comparison, we analyzed the correlation of serum MCP-1 with the level of alkaline phosphatase and liver fibrosis in a group of patients with NAFLD and AIH. In the NAFLD group, the mean activity of AP was 75.3 ± 16.7 U/L, 92% of patients showed few changes in liver tissue, and 8% had advanced fibrosis. In the AIH group, the mean activity of AP was 165.5 ± 120.2 U/L, 80% of patients showed few changes in liver tissue, and 20% had advanced fibrosis. In the group of NAFLD patients, we found no association between the serum concentration of MCP-1 and alkaline phosphatase levels—R = 0.13, *p* > 0.05. and only found a weak correlation between the serum concentration of MCP-1 and liver fibrosis—R = 0.20, *p* = 0.04. In the group of AIH patients, we observed an association between the serum concentration of MCP-1 and alkaline phosphatase levels—R = 0.25, *p* = 0.030, and also noted a weak correlation between the serum concentration of MCP-1 and liver fibrosis—R = 0.29, *p* = 0.02.

### 2.4. MCP-1 Concentration and PBC-Specific Antibodies

**Figure 7:**
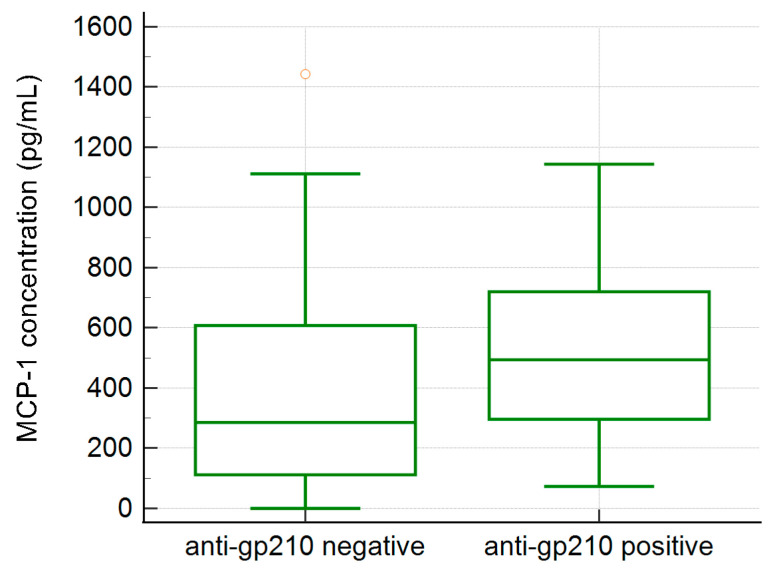
The MCP-1 concentrations in the anti-gp210 positive (561.2 ± 307.2 pg/mL) and anti-gp210 negative (342.7 ± 295.3 pg/mL) PBC patients, *p* = 0.0003.

We found a moderate positive correlation between serum MCP-1 concentration and AMA M2 levels in PBC patients (r = 0.36, *p* < 0.001), and no significant correlation between MCP-1 concentration and anti-gp210 antibody levels. However, we noticed a higher prevalence of patients with elevated MCP-1 levels in the tested anti-gp210 positive group of PBC patients 36/39 (92%) than in anti-gp210 negative PBC patients 43/81 (53%), *p* < 0.0001. The mean concentration of MCP-1 was also significantly higher in this group, 561.2 ± 307.2 vs. 342.7 ± 295.3 pg/mL, *p* = 0.0003 (Figure 7).

### 2.5. MCP-1 Concentration and the Survival of Patients

**Figure 8:**
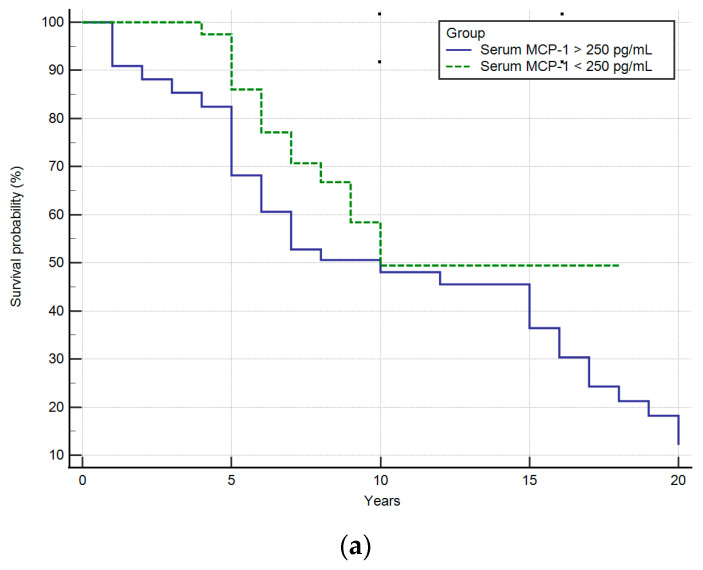

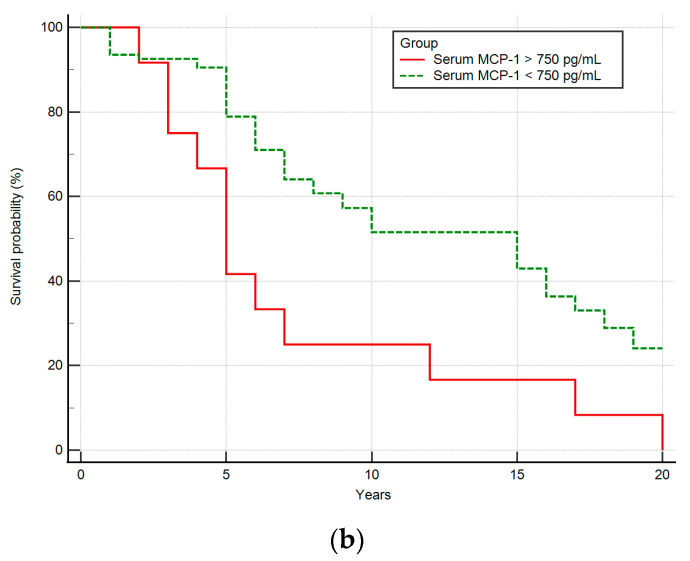
Kaplan–Meier curves demonstrating the survival of PBC patients with low and high serum MCP-1 concentration; (**a**) group of PBC patients with serum MCP-1 < 250 pg/mL and with serum MCP-1 > 250 pg/mL; (**b**) group of PBC patients with serum MCP-1 < 750 pg/mL and with serum MCP-1 > 750 pg/mL.

Analysis of the survival of patients who were positive and negative for serum MCP-1 (Figure 8) demonstrated that the high concentration of this chemokine correlated with the length of life or time to liver transplantation in PBC patients.

The survival time of patients or the period for liver transplantation was shorter for those with serum MCP-1 > 250 pg/mL, although it was not statistically significant, *p* = 0.073. In the group of patients with serum MCP-1 > 750 pg/mL, the survival time of patients or the period for liver transplantation was significantly shorter, *p* = 0.004.

## 3. Discussion

PBC is a chronic, incurable disease that leads to liver failure and, in its advanced form, to death. The risk of developing advanced cirrhosis of the liver is especially important in this regard. When looking at the effectiveness of treatment in slowing the progression of the disease, early diagnosis is crucial. For the final assessment and diagnosis, it is necessary to calculate the state of liver fibrosis and the risk of complications. Widely known and used algorithms, biomarkers, and their combinations, are not always well applied, and are not yet optimal as a screening test to diagnose progressed fibrosis in PBC. Although various biomarkers of fibrosis exist, the most promising circulating serum biomarker for PBC has not yet been identified.

An abnormal serum level of AP is characteristic in patients with PBC [8], and they may also have higher serum transaminase (aspartate aminotransferase (AST) and alanine aminotransferase (ALT)) activity. A high AST/ALT ratio is an indicator of ongoing liver fibrosis, and high GGT levels are often found before AP increases [8]. Hyperbilirubinemia occurs as PBC progresses, and significant increases are typical of the progressed disease. AMA or ANA (anty-gp210 autoantibodies) positivity is a strong marker of PBC in patients with uncharacteristic liver biochemistry. Diagnosis of PBC based on AMA or ANA reactivity is only possible when the presence of antibodies is associated with abnormal serum liver test results. In accordance with the actual EASL guidelines, a liver biopsy is not obligatory for the diagnosis of PBC, but it is significant when PBC-specific antibodies are absent [40]. Non-invasive biomarkers that would allow the diagnosis of patients at risk of advanced fibrosis and accompanying complications are now constantly being sought.

Several studies have documented the role of MCP-1 in liver diseases [35,36,37,38,39], including Zhou et al, who showed an increase in MCP-1 in primary sclerosing cholangitis patients (PSC) [41]. Slightly less is known about the role of MCP-1 in autoimmune liver disorders and MCP-1 serum concentrations. There is, for example, little information in the literature on the association between PBC and MCP-1 serum levels. Tsuneyama et al. showed that monocyte chemotactic proteins -1, -2 and-3 (MCP-1;2;3) are clearly expressed in portal tracts and granulomas in primary biliary cirrhosis [42]. Recently, Galluci et al. studied the anti-inflammatory mechanism of fenofibrate by inhibiting NF-kappa B signaling in human macrophages and clinical outcomes in patients with PBC and MCP-1 [43]. Analyzing non-autoimmune inflammatory liver diseases, and referring to the reports of obese patients with NAFLD showed that elevated serum levels of MCP-1 were also found in patients diagnosed with NAFLD [44]. Glass et al. also concluded that serum MCP-1 was associated with hepatic fibrosis in patients with histologically confirmed NAFLD [39]. A systematic review and network meta-analysis of chemokines in non-alcoholic fatty liver disease presented elevated concentrations of MCP-1 associated with NAFL or NASH [45]. Queck et al. found a relationship between circulating MCP-1 and the severity of liver disease [46]. Serum MCP-1 was a promising non-invasive tool for the diagnosis of NASH in the Egyptian population, confirming the role of MCP-1 in the pathogenesis of NASH [47]. This relationship between circulating MCP-1 and liver fibrosis in patients with NAFLD was only confirmed by a study by Ferrari-Cestari et al. [48] Conversely, Kobayashi et al. found a positive linear correlation between serum MCP-1 and type IV collagen that was statistically significant [19].

The main observation of our study was that circulating MCP-1 is correlated with the severity of liver cirrhosis. We noted that serum MCP-1 levels showed a statistically significant increase with increasing fibrosis severity in our PBC patients. The levels of MCP-1 were significantly associated with the presence of liver fibrosis. We also observed an association between the occurrence of higher concentrations of MCP-1 and higher levels of AP. De Munck et al. suggest that intestinal barrier dysfunction triggers the release of pro-inflammatory microbiological products derived from the intestines, and induce later inflammatory chemokines such as MCP-1, which are involved in the etiology of chronic liver disease [49]. An analogous situation may occur in PBC.

We showed that serum MCP-1 concentrations were significantly higher in patients with PBC, in comparison with healthy controls. The elevated MCP-1 level has been observed in other autoimmune diseases such as autoimmune connective tissue diseases. Pulito-Cueto et al. presented an association of MCP-1 with lung involvement in RA. Patients with RA and interstitial lung disease presented higher levels of MCP-1 [32]. MCP-1 serum levels in patients with pulmonary fibrosis of interstitial lung disease associated with rheumatoid arthritis were over 640 pg/mL, and in our PBC patients we observed a concentration above 400 pg/mL, which was also significantly higher than the control group. Serum MCP-1 levels also increased in polymyositis/dermatomyositis patients and were very often correlated with the complication of interstitial lung disease [32,34]. The upper concentration of MCP-1 in PBC can be affected by chronic systemic exposure to proinflammatory cytokines that are specific to PBC, as in RA [32,50].

We presented a comparison of the levels of different fibrosis markers: procollagen type III, hyaluronic acid, FIB-4 index, APRI, collagen type IV and circulating MCP-1 for different stages of the disease. Circulating MCP-1 demonstrated the better correlation(R = 0.68), although a good correlation was also observed among the FIB-4 index and stage of fibrosis(R = 0.59). Fujinaga et al. presented 10 serum parameters for the estimation of liver fibrosis and prediction of clinical outcomes in PBC [51]. Authors correlated histological stages based on both of the Scheuer and Nakanuma classifications with fibrosis indices. They found that an enhanced liver fibrosis (ELF) score had the highest correlation coefficient for liver fibrosis when evaluated with either classification. Contrary to our studies based on the Ludwig classifications, the correlation with FIB-4 was found to be very weak. Both in our work and in Fuinaga’s work, there was a very weak correlation between stage of fibrosis and the procollagen type III, hyaluronic acid and APRI, and practically no correlation between the stage of fibrosis and collagen type IV. Fujinaga et al. have not investigated the circulating MCP-1. We showed that for the group of patients with serum MCP-1 > 750 pg/mL the survival time of patients or the period for liver transplantation was significantly shorter. MCP-1 may therefore also have prognostic value in the course of the disease.

We compared the results for serum MCP-1 with immunological parameters. The level of MCP-1 in our group of PBC patients with AMA M2 and anti-gp210 antibodies has been evaluated, due to their high specificity in the diagnosis of PBC. We found a moderate positive correlation between serum MCP-1 concentration and AMA M2 levels and an increased concentration of MCP-1 in the ANA (anti-gp210 antibodies) positive group. This was an interesting finding, as previous work on PBC had shown that positive anti-gp210 antibodies are a marker of poor prognosis in PBC patients [52]. These autoantibodies were independently linked with a greater risk of liver-related death or transplantation [53,54].

In addition, measuring the level of MCP-1 could be important in the therapeutic approach in the future. Baeck et al. and Parker et al. reported that the genetic or pharmacological inhibition of monocyte recruitment via the MCP-1/CCR2 chemokine axis inhibited or decreased fibrosis in pre-clinical models of chronic liver disease [55,56]. They also confirmed that the MCP-1 level is a sensitive measure that can be used to estimate the therapeutic response of the anti-cytokine therapy [20], which could also offer a helpful solution in the case of PBC.

## 4. Materials and Methods

### 4.1. Patients

We collected serum samples from 120 patients (116 women, 4 men; median age: 51 years-of-age; age range: 28–73 years-of-age), who were diagnosed at the Centre of Postgraduate Medical Education (in Warsaw, Poland). The recognition of PBC was confirmed using generally accepted criteria, which corresponded to the practice guidelines of the European Association for The Study of Liver Diseases (EASL) for PBC [40]. A biopsy was performed on all patients, and it was found most patients had positive AMA antibodies. In AMA-negative PBC patients diagnosis was confirmed by antinuclear antibody positivity or by liver biopsy. We excluded patients with serum levels who tested positive for the hepatitis B surface antigen (HBsAg), anti-hepatitis A (IgM), and hepatitis C virus, and also excluded patients with alcoholism, and AIH (autoimmune hepatitis)/PBC overlap syndrome. The pathologic control group contained 40 patients (20 females, 20 males; median age: 45 years-of-age; age range: 19–67 years-of-age) with NAFLD; and 20 patients with autoimmune hepatitis—AIH (18 females, 2 males; median age: 48 years-of-age; age range: 22–68 years-of-age). Serum samples from 30 healthy adult blood donors (22 females, 8 males; median age: 33 years-of-age; age range: 19–53 years-of-age) were collected at the Warsaw Blood Bank.

The study protocol was conducted in accordance with the ethical guidelines of the Declaration of Helsinki and was approved by the the Centre of Postgraduate Medical Education’s ethical committee (Warsaw; approval number 71/PB/2019).

### 4.2. Detection of MCP-1

The MCP-1 level was evaluated by using a commercial ELISA kit (Boster Biological Technology, Pleasanton, CA, USA, Catalog #EZ0441), that corresponded to the manufacturer’s guidelines. Intra-assay and inter-assay performances were 3.1% and 5.3%, respectively. MCP-1 concentrations >250 pg/mL were considered positive. The cut-off value was determined by using the ROC curve.

### 4.3. Detection of Fibrosis Markers

Human procollagen type III concentration was evaluated by using a commercial ELISA kit (Causabio Technology LLC., Houston, TX, USA), Hyaluronic acid and collagen type IV concentration was evaluated by using a commercial ELISA kit (Aviva Systems Biology, San Diego, CA, USA). The FIB-4 index used the following formulas: FIB-4 index = (age × AST)/[(PLT count) × (ALT) 1/2]; APRI (AST to Platelet Ratio Index) = [(sample AST/reference AST)100]/PLT count.

### 4.4. Detection of Autoantibodies

Anti-gp210 antibodies and AMA M2 were determined by using commercially available ELISA kits (QUANTA Lite^®^ gp210; Inova Diagnostics, San Diego, CA, USA and QUANTA Lite^®^ M2 EP-MIT3, respectively; Inova Diagnostics, San Diego, CA, USA), according to the manufacturer’s instructions. The intra-assay performance of these kits was 4.6% and 2.9%, respectively, and their inter-assay performance was 5.8% and 6.1%, respectively.

### 4.5. Statistical Analysis

Prevalence rates were evaluated between groups by using the chi-square test and Fisher’s exact test. Continuous data were summarized as mean ± standard deviation (SD), and categorical data were summarized as frequencies. Continuous variables were evaluated using the Mann-Whitney test and were expressed as median ± interquartile range (IQR); *p* < 0.05 was considered statistically significant. All statistical analyses were presented using MedCal for Windows, version 7.4.1.0 (MedCal Software, Mariakerke, Belgium).

## 5. Conclusions

Circulating MCP-1 had a high correlation coefficient for liver fibrosis that was estimated according to Ludwig’s classification and showed a better correlation than other fibrosis markers. We found a correlation between MCP-1 and alkaline phosphatase, and also specific autoantibodies in PBC patients. Our results showed that the profibrogenic cytokine of monocyte, such as MCP-1, could take place in the progression of liver fibrosis in the PBC. Serum MCP-1 concentrations suggest that the circulating MCP-1 levels could be potential noninvasive biomarkers for liver fibrosis connected with PBC. These results can be important for clinical practice addressed to the diagnosis and assessment of the stage of fibrosis. New combinations of biomarkers associated with various aspects of the disease could support the diagnosis of PBC.

## Figures and Tables

**Table 1:** Characteristic of PBC patients and healthy control groups.

		Primary Biliary Cholangitis Patients (*n* = 120)	Healthy Adult Blood Donors (*n* = 30)	*p* Value
Age, years	Age, years	51 (15)	33 (12)	
Females/males	Females/males	116/4	22/8	
Bilirubin (Total), mg/dL	Bilirubin (Total), mg/dL	2.1 (2.0)	0.7 (0.6)	0.0002
AST, U/L	AST, U/L	85.5 (53.3)	22.5 (21.6)	<0.0001
ALT, U/L	ALT, U/L	93.8 (72.8)	15.1 (26.2)	<0.0001
AP, U/L	AP, U/L	301.0 (228.9)	38.7 (16.8)	<0.0001
γ-GT, U/L	γ-GT, U/L	299.4 (270.2)	18.6 (4.8)	<0.0001
Albumin (g/dL)	Albumin (g/dL)	3.5 (1.0)	4.5 (2.3)	0.0004
γ-globulin (g/dL)	γ-globulin (g/dL)	1.8 (1.2)	1.1 (0.2)	0.0018
AMA M2	AMA M2	104 (87%)	0 (0%)	<0.0001
Anti-gp210 antibodies	Anti-gp210 antibodies	39 (33%)	0 (0%)	0.0003
Early histological stage (I/II)	Early histological stage (I/II)	75 (63%)	-	
Advanced histological stage (III/IV)	Advanced histological stage (III/IV)	40 (33%)	-	
Ambiguous histological stage	Ambiguous histological stage	5 (4%)	-	

Data are presented as mean (SD). Abbreviations: γ-GT, γ-glutamyl transpeptidase; ALT, alanine aminotransferase; AP, alkaline phosphatase; AST, aspartate aminotransferase. Conversion factors to SI units are as follows: for bilirubin, 17.1; for AST, ALT, AP, and γ-GT, 0.0167.

## Data Availability

The raw data supporting the article conclusions will be made available by the authors, without undue reservation.

## References

[1] Carey E.J., Ali A.H., Lindor K.D. (2015). Primary Biliary Cirrhosis. Lancet.

[2] Zhang Q., Liu Z., Wu S., Duan W., Chen S., Ou X., You H., Kong Y., Jia J. (2019). Meta-Analysis of Antinuclear Antibodies in the Diagnosis of Antimitochondrial Antibody-Negative Primary Biliary Cholangitis. Gastroenterol. Res. Pract..

[3] Trivella J., John B.V., Levy C. (2023). Primary biliary cholangitis: Epidemiology, prognosis, and treatment. Hepatol. Commun..

[4] Hirschfield G.M., Chazouillères O., Cortez-Pinto H., Macedo G., de Lédinghen V., Adekunle F., Carbone M. (2021). A Consensus Integrated Care Pathway for Patients with Primary Biliary Cholangitis: A Guideline-Based Approach to Clinical Care of Patients. Expert Rev. Gastroenterol. Hepatol..

[5] You H., Ma X., Efe C., Wang G., Jeong S.-H., Abe K., Duan W., Chen S., Kong Y., Zhang D. (2022). APASL Clinical Practice Guidance: The Diagnosis and Management of Patients with Primary Biliary Cholangitis. Hepatol. Int..

[6] Tian S., Hu Y., Zhang M., Wang K., Guo G., Li B., Shang Y., Han Y. (2023). Integrative bioinformatics analysis and experimental validation of key biomarkers for risk stratification in primary biliary cholangitis. Arthritis Res. Ther..

[7] Wang M., Jin Y., Xu A. (2023). Diagnostic and prognostic value of quantitative detection of antimitochondrial antibodies subtype M2 using chemiluminescence immunoassay in primary biliary cholangitis. CCLM.

[8] Lindor K.D., Bowlus C.L., Boyer J., Levy C., Mayo M. (2019). Primary Biliary Cholangitis: Practice Guidance from the American Association for the Study of Liver Diseases. Hepatology.

[9] Granito A., Muratori L., Tovoli F., Muratori P. (2021). Autoantibodies to Speckled Protein Family in Primary Biliary Cholangitis. Allergy Asthma Clin. Immunol..

[10] Ozaslan E., Efe C., Ozaslan N.G. (2016). The Diagnosis of Antimitochondrial Antibody-Negative Primary Biliary Cholangitis. Clin. Res. Hepatol. Gastroenterol..

[11] Nakamura M. (2014). Clinical significance of autoantibodies in primary biliary cirrhosis. Semin. Liver Dis..

[12] Yamagiwa S., Kamimura H., Takamura M., Aoyagi Y. (2014). Autoantibodies in primary biliary cirrhosis: Recent progress in research on the pathogenetic and clinical significance. World J. Gastroenterol..

[13] Bauer A., Habior A., Wieszczy P., Gaweł D. (2021). Analysis of Autoantibodies Against Promyelocytic Leukemia Nuclear Body Com-Ponents and Biochemical Parameters in Sera of Patients with Primary Biliary Cholangitis. Diagnostics.

[14] Janmohamed A., Trivedi P.J. (2018). Patterns of disease progression and incidence of complications in primary biliary cholangitis (PBC). Best. Pract. Res. Clin. Gastroenterol..

[15] Bauer A., Habior A., Gawel D. (2022). Diagnostic and Clinical Value of Specific Autoantibodies against Kelch-like 12 Peptide and Nuclear Envelope Proteins in Patients with Primary Biliary Cholangitis. Biomedicines.

[16] Roeb E. (2020). Matrix Metalloproteinases and Liver Fibrosis (Translational Aspects). Matrix Biol..

[17] Bauer A., Habior A. (2022). Concentration of Serum Matrix Metalloproteinase-3 in Patients with Primary Biliary Cholangitis. Front. Immunol..

[18] Cao S., Liu M., Sehrawat T.S., Shah V.H. (2021). Regulation and functional roles of chemokines in liver diseases. Nat. Rev. Gastroenterol. Hepatol..

[19] Kobayashi K., Yoshioka T., Miyauchi J., Nakazawa A., Yamazaki S., Ono H., Tatsuno M., Iijima K., Takahashi C., Okada Y. (2017). Monocyte Chemoattractant Protein-1 (MCP-1) as a Potential Therapeutic Target and a Noninvasive Biomarker of Liver Fibrosis Associated with Transient Myeloproliferative Disorder in Down Syndrome. J. Pediatr. Hematol. Oncol..

[20] Singh S., Anshita D., Ravichandiran V. (2021). MCP-1: Function, regulation, and involvement in disease. Int. Immunopharmacol..

[21] Deshmane S.L., Kremlev S., Amini S., Sawaya B. (2009). EMonocyte chemoattractant protein-1 (MCP-1): An overview. J. Interferon Cytokine Res..

[22] Hasegawa M., Sato S., Takehara K. (1999). Augmented production of chemokines (monocyte chemotactic protein-1 (MCP-1), macrophage inflammatory protein-1α (MIP-1α) and MIP-1β) in patients with systemic sclerosis: MCP-1 and MIP-1α may be involved in the development of pulmonary fibrosis. Clin. Exp. Immunol..

[23] Taghavi Y., Hassanshahi G., Kounis N.G., Koniari I., Khorramdelazad H. (2019). Monocyte chemoattractant protein-1 (MCP-1/CCL2) in diabetic retinopathy: Latest evidence and clinical considerations. J. Cell Commun. Signal..

[24] Yoshimura T., Li C., Wang Y., Matsukawa A. (2023). The chemokine monocyte chemoattractant protein 1/CCL2 is a promoter of breast cancer metastasis. Cell Mol. Immunol..

[25] Lin J., Kakkar V., Lu X. (2014). Impact of MCP-1 in atherosclerosis. Curr. Pharm. Des..

[26] Kim M.J., Tam F.W.K. (2011). Urinary monocyte chemoattractant protein-1 in renal disease. Clin. Chim. Acta.

[27] Li X., Huang Y., Liu Y., Yan S., Li L., Cheng L., Li H., Zhan H., Zhang F., Li Y. (2023). Circulating VEGF-A, TNF-α, CCL2, IL-6, and IFN-γ as biomarkers of cancer in cancer-associated anti-TIF1-γ antibody-positive dermatomyositis. Clin. Rheumatol..

[28] An Z., Qin J., Bo W., Li H., Jiang L., Li X., Jiang J. (2022). Prognostic Value of Serum Interleukin-6, NF-κB plus MCP-1 Assay in Patients with Diabetic Nephropathy. Dis. Markers.

[29] Kaushansky N., Bakos E., Becker-Herman S., Shachar I., Ben-Nun A. (2019). Circulating Picomolar Levels of CCL2 Downregulate Ongoing Chronic Experimental Autoimmune Encephalomyelitis by Induction of Regulatory Mechanisms. J. Immunol..

[30] Iwamoto H., Izumi K., Nakagawa R., Toriumi R., Aoyama S., Shimada T., Kano H., Makino T., Kadomoto S., Yaegashi H. (2022). Usefulness of serum CCL2 as prognostic biomarker in prostate cancer: A long-term follow-up study. Jpn. J. Clin. Oncol..

[31] Yalçinkaya Y., Çinar S., Artim-Esen B., Kamali S., Öcal L., Deniz G., Inanç M. (2016). The relationship between vascular biomarkers and disease characteristics in systemic sclerosis: Elevated MCP-1 is associated with fibrotic manifestations. Clin. Exp. Rheumatol..

[32] Pulito-Cueto V., Remuzgo-Martínez S., Genre F., Atienza-Mateo B., Mora-Cuesta V.M., Iturbe-Fernández D., Lera-Gómez L., Mora-Gil M.S., Prieto-Peña D., Portilla V. (2022). Elevated VCAM-1, MCP-1 and ADMA serum levels related to pulmonary fibrosis of interstitial lung disease associated with rheumatoid arthritis. Front. Mol. Biosci..

[33] Schmidt K., Martinez-Gamboa L., Meier S., Witt C., Meisel C., Hanitsch L.G., Becker M.O., Huscher D., Burmester G.R., Riemekasten G. (2009). Bronchoalveoloar lavage fluid cytokines and chemokines as markers and predictors for the outcome of interstitial lung disease in systemic sclerosis patients. Arthritis Res. Ther..

[34] Wu Ch Y., Li L., Zhang L.H. (2019). Detection of serum MCP-1 and TGF-β1 in polymyositis/dermatomyositis patients and its significance. Eur. J. Med. Res..

[35] Marsillach J., Bertran N., Camps J., Ferré N., Riu F., Tous M., Coll B., Alonso-Villaverde C., Joven J. (2005). The role of circulating monocyte chemoattractant protein-1 as a marker of hepatic inflammation in patients with chronic liver disease. Clin. Biochem..

[36] Haukeland W.J., Damås K., Konopski Z., Birkeland K., Bjøro K., Aukrust P. (2006). Systemic inflammation in nonalcoholic fatty liver disease is characterized by elevated levels of CCL2. J. Hepatol..

[37] Kazmi N., Wallen G.R., Yang L., Alkhatib J., Schwandt M.L., Feng D., Gao B., Diazgranados N., Ramchandani V.A., Barb J.J. (2022). An exploratory study of pro-inflammatory cytokines in individuals with alcohol use disorder: MCP-1 and IL-8 associated with alcohol consumption, sleep quality, anxiety, depression, and liver biomarkers. Front. Psychiatry.

[38] Ajmera V., Perito E.R., Bass N.M., Terrault N.A., Yates K.P., Gill R., Loomba R., Diehl A.M., Aouizerat B.E., for the NASH Clinical Research Network (2017). Novel Plasma Biomarkers AssociatedWith Liver Disease Severity in Adults with Nonalcoholic Fatty Liver Disease. Hepatology.

[39] Glass O., Henao R., Patel K., Guy C.D., Gruss H.J., Syn W., Moylan C.A., Streilein R., Hall R., Diehl A.M. (2018). Serum Interleukin-8, Osteopontin, and Monocyte Chemoattractant Protein 1 are Associated With Hepatic Fibrosis in Patients With Nonalcoholic Fatty Liver Disease. Hepatol. Commun..

[40] European Association for the Study of the Liver (2017). EASL Clinical Practice Guidelines: The diagnosis and management of patients with primary biliary cholangitis. J. Hepatol..

[41] Zhou T., Bartelheimer K., Ruping F., Rupp C., Sauer P., Koschny R., Mehrabi A., Mieth M., von Haken R., Weiss K.H. (2020). Intrahepatic biliary strictures after liver transplantation are morphologically similar to primary sclerosing cholangitis but immunologically distinct. Eur. J. Gastroenterol. Hepatol..

[42] Tsuneyama K., Harada K., Yasoshima M., Hiramatsu K., Mackay C.R., Mackay I.R., Gershwin M.E., Nakanuma Y. (2001). Monocyte chemotactic protein-1,-2, and-3 are distinctively expressed in portal tracts and granulomata in primary biliary cirrhosis: Implications for pathogenesis. J. Pathol..

[43] Gallucci G.M., Alsuwayt B., Auclair A.M., Boyer J.L., Assis D.N., Ghonem N.S. (2022). Fenofibrate Downregulates NF-kappa B Signaling to Inhibit Pro-inflammatory Cytokine Secretion in Human THP-1 Macrophages and During Primary Biliary Cholangitis. Inflammation.

[44] Kirovski G., Dorn C., Huber H., Moleda L., Niessen C., Wobser H., Schacherer D., Buechler C., Wiest R., Hellerbrand C. (2011). Elevated systemic monocyte chemoattractant protein-1 in hepatic steatosis without significant hepatic inflammation. Exp. Mol. Pathol..

[45] Pan X., Kaminga ACh Liu A., Wen S.W., Chen J., Luo J. (2020). Chemokines in non-alcoholic fatty liver disease: A systematic review and network meta-analysis. Front. Immunol..

[46] Queck A., Bode H., Uschner F.E., Brol M.J., Graf C., Schulz M., Jansen C., Praktiknjo M., Schierwagen R., Klein S. (2020). Systemic MCP-1 Levels Derive Mainly from Injured Liver and are Associated with Complications in Cirrhosis. Front. Immunol..

[47] Ali A.A., Fouda A., Abdelaziz E.S., Abdelkawy K., Ahmed M.H. (2021). The promising role of CCL2 as a noninvasive marker for nonalcoholic steatohepatitis diagnosis in Egyptian populations. Eur. J. Gastroenterol. Hepatol..

[48] Ferrari-Cestari M., Ferrari-Cestari M., Okano S., Okano S., Patel P.J., Patel P.J., Horsfall L.U., Horsfall L.U., Keshvari S., Keshvari S. (2023). Serum CC-Chemokine Ligand 2 is Associated with Visceral Adiposity but not Fibrosis in Patients with Non-Alcoholic Fatty Liver Disease. Dig. Dis..

[49] De Munck T.J.I., Xu P., Verwijs H.J.A., Masclee A.A.M., Jonkers D., Verbeek J., Koek G.H. (2020). Intestinal permeability in human nonalcoholic fatty liver disease: A systematic review and meta-analysis. Liver Int..

[50] Rantapää-Dahlqvist S., Boman K., Tarkowski A., Hallmans G. (2007). Up-regulation of monocyte chemoattractant protein-1 expression in anti-citrulline antibody and immunoglobulin M rheumatoid factor positive subjects precede the onset of inflammatory response and development of overt rheumatoid arthritis. Ann. Rheum. Dis..

[51] Fujinaga Y.M., Namisaki T., Takaya H., Tsuji Y., Suzuki J., Shibamoto A., Kubo T., Iwai S., Tomooka F., Takeda S. (2021). Enhanced liver fibrosis score as a surrogate of liver-related complications and mortality in primary biliary cholangitis. Medicine.

[52] Haldar D., Janmohamed A., Plant T., Davidson M., Norman H., Russell E., Serevina O., Chung K., Qamar K., Gunson B. (2020). Antibodies to gp210 and understanding risk in patients with primary biliary cholangitis. Liver Int..

[53] Chen Q., Zhong R., Dong K., Wang Y., Kui Y., Ma B., Wen X., Jin Q. (2022). The prognostic value of antibodies to gp210 among patients with primary biliary cholangitis in Northeast China. Dig. Liver Dis..

[54] Cristoferi L., Gerussi A., Invernizzi P. (2021). Anti-gp210 and other anti-nuclear pore complex autoantibodies in primary biliary cholangitis: What we know and what we should know. Liver Int..

[55] Baeck C., Wehr A., Karlmark K.R., Heymann F., Vucur M., Gassler N., Huss S., Klussmann S., Eulberg D., Luedde T. (2012). Pharmacological inhibition of the chemokine CCL2 (MCP-1) diminishes liver macrophage infiltration and steatohepatitis in chronic hepatic injury. Gut.

[56] Parker R., Weston C.J., Miao Z., Corbett C., Armstrong M.J., Ertl L., Ebsworth K., Walters M.J., Baumart T., Newland D. (2018). CC chemokine receptor 2 promotes the recruitment of myeloid cells associated with insulin resistance in nonalcoholic fatty liver disease. Am. J. Physiol. Gastrointest. Liver Physiol..

